# Editorial to the Special Issue “Molecular Motors: From Single Molecules to Cooperative and Regulatory Mechanisms In Vivo”

**DOI:** 10.3390/ijms23126605

**Published:** 2022-06-14

**Authors:** Marco Capitanio, Massimo Reconditi

**Affiliations:** 1 Department of Physics and Astronomy, University of Florence, 50019 Florence, Italy; marco.capitanio@unifi.it; 2 LENS—European Laboratory for Non-Linear Spectroscopy, 50019 Florence, Italy; 3 Department of Experimental and Clinical Medicine, University of Florence, 50134 Florence, Italy; 4 PhysioLab, University of Florence, 50019 Florence, Italy

The Molecular motors or motor proteins are able to generate force and do mechanical work that is used to displace a load or produce relative movements between molecules or macromolecular assembles. The energy is supplied by the hydrolysis of ATP (or GTP in some cases), and the way a motor transforms chemical energy into mechanical output il largely unknown. Because of their many fundamental biological functions, molecular motors are ubiquitous in living cells: to cover but a few of these functions, they are responsible for cell movement and division, driving intracellular trafficking inside the cell, and can work cooperatively to produce macroscopic outputs such as in the case of muscle contraction or bending movement in flagella and cilia. Linear motors, to accomplish their tasks, move along a track. Myosin moves along actin filaments, kinesin and dynein along microtubules, RNA polymerase, ribosome and chromatin remodelers along nucleic acid tracks [1,2,3,4,5].

One of the most studied molecular motor is the class II myosin that is responsible for muscle contraction. This is reflected in the distribution of the papers published in this Special Issue, where three out of six are about the molecular motor in muscle.

Muscle myosin II is a dimeric non processive motor. In the structural unit of striated muscle, both skeletal and cardiac, myosin motors are arranged on the thick filament in two opposite arrays of ca 300 motors that work in parallel and produce force during their interaction with the overlapping actin filaments. The contractile proteins myosin and actin are accompanied by a plethora of other sarcomeric proteins with both structural and regulatory roles.

The paper by Månsson published in this SI [6] shows supports to the hypothesis that it is possible to model the mechanical properties of muscle extrapolating at the level of the motor arrays the properties of single molecule of both myosin, the motor, and actin, the track, despite the complex environment in the sarcomere. Since it is well known the regulatory role of at least the thin filament proteins troponin and tropomyosin in the activation of muscle contraction, Månsson hypothesis refers to the fully activated state at saturating intracellular Ca^2+^. Also alternative views are discussed, mentioning a possible regulatory role for the thick filament protein MyBP-C or the strain on the thick filament itself that mechanically couples the myosin motors. To solve the question, future tests of the hypothesis are discussed.

The paper by Pertici and co-workers [7] presents a methodological improvement of a synthetic myosin II-based nanomachine that allows the study of the emergent properties of the array arrangement of the myosin motors on the meso-scale. In the nanomachine, a bed of heavy-meromyosin fragments (HMM) carried on a piezoelectric nanopositioner is brought to interact with an actin filament attached with the proper polarity to a bead trapped by dual laser optical tweezers. The device promises to provide answer, among other applications, to the questions posed by Månsson. In fact, it would be possible to add/remove specific protein components one at the time to/from the essential structure of the sarcomere reconstituited in the nanomachine and study their role in the emergent properties of the motor array.

Somewhat challenging Månsson’s hypothesis, the contribution by Marcucci and co-workers [8] shows on the basis of their experimental results interpreted with a 3D model of the half-sarcomere that the geometric hindrance and biased motor detachment from actin would generate filament sliding without structural change in the motor, like the rotation of the lever arm. Rotation of the lever arm is generally believed to be the way that a myosin motor uses to displace the actin filament relative to its support, either the thick filament in situ or the functionalised glass surface in the in vitro motility assay [9,10,11]. In the model proposed by Marcucci and co-workers the structural change is integrated in a Brownian-ratchet framework and takes into account of the recently discovered mechanosensing mechanism for the recruitment of the myosin motors [12,13].

Other two papers of the SI deal with the kinesin motor. In cell, the kinesin motors are in their dimeric form, and the way they walk along a microtubule (MT, the track) is explained by a hand-over-hand mechanism [14].

The research article by Mizuhara and Takano [15] is aimed at elucidating the mechanism of the biased Brownian motion along the MT of the kinesin-3 type KIF1A, which is able to move along the MT unidirectionally in its monomeric form [16]. For that purpose, the two authors perform molecular dynamics simulations considering a minimal system constituted by a single monomeric KIF1A and a single MT protofilament and provide a mechanism for the motion. Whether this mechanism is at work also in the cell, where the dimeric form of KIF1A exerts its function of cargo transporter, is left as an open question.

In the other paper on the kinesin motors, Qin and colleagues [17] review the studies on mechanochemical coupling responsible for the motion of conventional kinesin-1 motor. The framework of their review is that of the hand-over-hand mechanism and the key question is the way the chemical energy released by an ATP molecule is exploited to produce mechanical work, i.e., displacement and force generation. The regulatory role of the MT in the cycle, apart its function as the track, is also discussed. The authors conclude that while the walking mechanism and mechanochemical coupling of kinesin-1 is has been largely elucidated, the detailed mechanisms that regulate ATP binding and ADP release are still unclear.

A different class of motors, the chromatin remodelers, are responsible for restructure, mobilize or eject nucleosomes in the chromatin, which play an important role in the epigenetic regulation of gene expression. Chromatin remodelers may be monomeric enzymes or multiprotein complex. The activity of these motors is reviewed in the contribution by Morgan and colleagues [18]. As for the other classes of motors, models for the mechanism of their action are proposed based either on structural changes producing a power-stroke or ratchets that bias the thermal motion of nucleosomes. Future works will help to discriminate between the two models of action.
